# Sperm morphology and the evolution of intracellular sperm–egg interactions

**DOI:** 10.1002/ece3.4027

**Published:** 2018-04-24

**Authors:** Helen M. Southern, Mitchell A. Berger, Philippe G. Young, Rhonda R. Snook

**Affiliations:** ^1^ Department of Animal and Plant Sciences University of Sheffield Sheffield UK; ^2^ Mathematics University of Exeter Exeter UK; ^3^ College of Engineering, Mathematics, and Physical Sciences University of Exeter Exeter UK; ^4^ Department of Zoology Stockholm University Stockholm Sweden

**Keywords:** Drosophila, fertilization, reproductive isolation, speciation, sperm–egg interactions

## Abstract

Sperm morphology is incredibly diverse, even among closely related species, yet the coevolution between males and females of fertilization recognition systems is necessary for successful karyogamy (male and female pronuclear fusion). In most species, the entire sperm enters the egg during fertilization so sperm morphological diversity may impact the intracellular sperm–egg interactions necessary for karyogamy. We quantified morphological variation of sperm inside eggs prior to and following karyogamy in several species of *Drosophila* to understand whether evolution of sperm morphology could influence intracellular sperm–egg interactions (ISEIs). We measured seven parameters that describe ISEIs among species to determine whether these parameters varied both within a species across development and across species at the same developmental stage. We used heterospecific crosses to test the relative role of male origin, female origin, and interaction between the male and female in determining ISEIs. We found that sperm shape changed within a species as development proceeded and, at particular development stages, species varied in some ISEIs. Parental origin had an effect on some ISEIs, with a general trend for a stronger female effect. Overall, our findings identify conserved and variable ISEIs among species and demonstrate the potential to contribute understanding to gamete evolution and development.

## 1 INTRODUCTION

Fertilization comprises complex morphological, physiological, and biochemical interactions between the gametes. Fertilisation will occur when these interactions are coordinated and coevolved between the sexes and be impaired when they are not. Thus, sex‐specific changes in the fertilisation environment may result in reproductive incompatibility between noncoevolved individuals (Rowe et al., 2015). Reproductive genes, some associated with sperm–egg recognitions systems, are known to evolve faster than nonreproductive genes (Clark, Aagaard, & Swanson, 2006; Swanson & Vacquier, 2002), such as in the lysin/VERL system of *Haliotis* abalone species (Panhuis, Clark, & Swanson, 2006). In this system, the sperm protein (lysin) functions in gamete recognition and creates a hole in the egg envelope during their interaction. Species‐specific changes in lysin appear to be rapidly driven by strong, positive selection (Hellberg & Vacquier, 1999; Vacquier, Swanson, & Lee, 1997; Yang, Swanson, & Vacquier, 2000), and this has been suggested to be an adaptive response to evolution in the vitelline envelope receptor for lysin (VERL) to which it binds (Swanson & Vacquier, 1998). This binding is species‐specific and prevents males from other species being able to fertilize a female's eggs. Rapid, adaptive protein evolution has also been demonstrated in mammals, where zona pellucida egg coat proteins (ZP2 and ZP3), which bind sperm to initiate the acrosome reaction, show d*N*/d*S* ratios greater than one (Swanson, Clark, Waldrip‐Dail, Wolfner, & Aquadro, 2001).

These studies show rapid evolution of gametic surface interactions (syngamy). However, perhaps the most morphologically diverse cell type is that of sperm, despite its conserved function of restoring diploidy to the next generation (Pitnick, Wolfner, & Suarez, 2009).

Sperm flagellum length differs between species in many taxa (Pitnick et al., 2009), and for many species, the entire sperm, including the flagellum, enters the egg during fertilization (Karr, Swanson, & Snook, 2009). Because sperm are acting within the egg cytoplasm, these interactions have been referred to as intracellular sperm–egg interactions (ISEIs; Snook, Chapman, Moore, Wedell, & Crudgington, 2009). Sperm entry into the egg at fertilisation has been shown to facilitate a variety of functions required for successful maternal and paternal pronuclear fusion (karyogamy) and early embryogenesis (Krawetz, 2005; Loppin, Dubruille, & Horard, 2015; Snook et al., 2009).

For example, in most organisms, a large component of the flagellum enters the egg and remains attached to the zygotic nucleus during development (Sutovsky & Schatten, 1999) and throughout early embryogenesis in *Drosophila* (Karr, 1996; Karr & Pitnick, 1996). At least a portion of the flagellum must be present to elicit sperm aster formation and karyogamy in many organisms as a consequence of the inclusion of paternal centrioles that are closely attached to the flagellum (Moomjy, Colombero, Veeck, Rosenwaks, & Palermo, 1999; Sutovsky & Schatten, 1999). Moreover, once inside the egg, there is an association between sterility and apparent abnormal conformation of the coiling structure of sperm flagellum within the egg cytoplasm (Lassy & Karr, 1996; Ohsako, Hirai, & Yamamoto, 2003). For example, *Drosophila* sperm are normally located in the anterior portion of the egg and take on an hypothesized species‐specific coiling structure within the egg (Karr, 1991). This 3D conformation likely appears only upon fertilization as no similar structuring has been described when sperm are in the female sperm storage organs prior to fertilization (Manier et al., 2010). Disruptions to these patterns are associated with lower karyogamy success, as in *Drosophila* misfire (*mfr*) mutants in which sperm are not located in the anterior portion of the egg and adopt a disrupted coiling structure compared to wild‐type sperm (Ohsako et al., 2003) and in crosses between different *D. melanogaster* populations that result in sterility (Alipaz, Wu, & Karr, 2001). Sperm also contribute various components to the oocyte that are necessary for early embryogenesis. For example, sperm in some species determine embryo polarity (Danilchik & Black, 1988; Gray et al., 2004; Pedersen, 2001; Piotrowska & Zernicka‐Goetz, 2001) and protein and RNA components, such as clusterin, may contribute to pronuclear formation (Ostermeier, Miller, Huntriss, Diamond, & Krawetz, 2004). The intracellular interactions between the sperm and egg cytoplasm are also important for successful karyogamy (Brent, MacQueen, & Hazelrigg, 2000; Loppin, Docquier, Bonneton, & Couble, 2000). For example, the *Drosophila* maternal mutation, *wispy* (wsy), prevents the proper configuration of pronuclei for karyogamy and early embryogenesis by stopping the female pronucleus migrating toward the male pronucleus (Brent et al., 2000).

Thus, given that sperm flagella rapidly evolve, such changes are predicted to impact ISEIs. Moreover, rapid sperm evolution should negatively impact ISEIs that could occur between species (analogous to the rapid evolution of sperm–egg interactions at the gamete surface). In many examples, sperm and eggs from different evolutionary lineages do not interact past the egg surface. However, in some cases, gametes may correctly interact during syngamy but may not subsequently result in karyogamy. For example, crosses between some geographical strains of *D. melanogaster* result in a low percentage of fertilized eggs because either the entire sperm does not enter the egg, sperm adopt an abnormal coiling structure within the egg, or the sperm is not restricted to the anterior portion of the egg as is normal for successful karyogamy (Alipaz et al., 2001).

While it is clear that ISEIs are critical to karyogamy and early embryonic development, there remain outstanding questions. It is unknown, the extent to which ISEIs vary within a species, whether ISEIs vary between species, and whether these interactions are primarily driven by either the sperm, the egg, or an interaction between sperm and egg. Here, we begin to answer these questions by developing a methodology for quantifying several gamete parameters to describe ISEIs during karyogamy and early development in Drosophila *obscura* group species. We use *Drosophila* because there is qualitative evidence that ISEIs change across development and between species (Karr, 1991, 1996; Karr & Pitnick, 1996; Pitnick & Karr, 1998; Snook & Karr, 1998), and sperm length varies considerably between species (Pitnick, Markow, & Spicer, 1995; Pitnick, Spicer, & Markow, 1995; Snook, 1997). We use closely related species from the *Drosophila obscura* group because they have relatively short sperm compared with other *Drosophila* species, which simplifies quantification of sperm shape. *Drosophila* is also a useful model to study ISEIs because matings between species—required to study putative negative impacts of rapid evolution of sperm morphology—are relatively easy to stage. We capitalize on the ability to successfully cross different *obscura* species, which allows assigning the contribution of sperm and egg, and their interaction, on ISEIs. Finally, ISEIs may be a major form of selection in *Drosophila*, providing greater scope to identify variation between species. We suggest this because, unlike marine invertebrates and mammals (Okabe, 2013; Santella, Vasilev, & Chun, 2012), there is no *fusion* between gametes at the surface. Instead, while *Drosophila* possesses an acrosome, it remains intact as it passes through the micropyle into the egg cytoplasm (Dudkina, Voronin, & Kiseleva, 2004; Perotti, 1975). Only once inside the egg cytoplasm do acrosome proteins function, but in this case to break down the sperm plasma membrane without exocytosis. Failure to breakdown the plasma sperm head membrane inside the egg results in male sterility (Wilson, Fitch, Bafus, & Wakimoto, 2006), which mimics fertilization failure of other taxa in which abnormalities in *surface* exocytic events result in sterility. The Drosophila acrosome is released into the egg cytoplasm and stays intact until at least prometaphase of the first embryonic division (Wilson et al., 2006). Overall, there is extensive scope for *Drosophila* ISEIs to play a similar role to that of gamete surface interactions in other taxa which are known to be under strong selection (Wilburn & Swanson, 2016).

## 2 MATERIALS AND METHODS

### 2.1 Fly species and maintenance

Four species from the *obscura* group were used for this experiment. *Drosophila pseudoobscura pseudoobscura* (hereafter abbreviated, Pse); a subspecies of *D. pseudoobscura* –*D. ps. bogotana* (Bog); the sister species, *D. persimilis* (Per); and the outgroup species, *D. miranda* (Mir) (Kulathinal, Stevison, & Noor, 2009). Flies were maintained in vials containing cornmeal‐agar‐molasses food media with added live yeast and housed at 22°C with a 12L:12D cycle. Experimental males and females were collected and sexed upon eclosion and housed in single‐sex yeasted food vials, 10 individuals per vial. We performed both conspecific (same species; coevolved ISEIs) and reciprocal heterospecific crosses (different species; noncoevolved ISEIs). When discussing specific heterospecific responses, we list the species of the female first.

### 2.2 Egg collection and staining

Collected virgin males and females were transferred to egg collection chambers at reproductive maturity (Snook, Markow, & Karr, 1994) in groups of 30. Males and females were either from the same species or from different species. When heterospecific, we performed reciprocal crosses. Each egg collection chamber was fitted with an egg‐laying (molasses/agar) plate, with added live yeast, for females to oviposit (Snook et al., 1994). After 24 hr, the first egg collection plate was discarded and replaced with a fresh one every hour. Eggs were harvested immediately from egg collection plates and dechorionated in 50% commercial bleach (active ingredient, 2.5% sodium hypochlorite) and 50% dH_2_O, rinsed in detergent (0.1% Triton X‐100) and then fixed in a 1:1 methanol:heptane solution. Fixed eggs were rinsed in methanol, followed by TBST, blocked with BSA, and then, the sperm inside eggs visualized by indirect immunofluorescence using an antiserum generated from rats immunized with *D. pseudoobscura* testes (Snook et al., 1994). Nuclei and polar bodies were made visible by nuclear staining with the DNA‐specific DAPI (4′,6‐diamidino‐2‐phenylindole). Approximately, 100 eggs were mounted onto standard microscope slides in 80% glycerol/PBS and sealed.

### 2.3 Image processing and image analysis

Confocal laser scanning microscopy was used to acquire images of antibody‐labeled eggs (Snook & Karr, 1998). We captured images of fluorescently labeled sperm within the egg and DAPI‐labeled sperm and egg pronuclei and subsequent nuclei after karyogamy as described previously (Snook & Karr, 1998). Images were standardized by capturing a 2D image every 2 μm through the z‐stack of the egg. The developmental stage of each egg was determined from DAPI labeling and counting the number of polar bodies and/or nuclei present. *Drosophila* development is syncytial. The zygote nucleus undergoes eight mitotic divisions within the central portion of the egg, and then nuclei migrate to the egg periphery and continue to divide. Up to the 14th mitotic division, all the nuclei share a common cytoplasm, and material can diffuse throughout the embryo. During this time, while no cell membranes exists other than that of the egg itself, the cytoplasm is not uniform. Each nucleus within the syncytium is surrounded by its own cytoskeletal proteins (Karr & Alberts, 1986). Here, we limit our analysis to the early rounds of nuclear division without cytokinesis as the number of nuclei are both restricted, contained within the central portion of the egg, and effects of both paternal and maternal gamete components can easily interact before cellularization. This simplifies measuring ISEIs. We imaged eggs at the pronuclear stage (PN) before karyogamy, 2N (one mitotic division), 4N (two mitotic divisions), and 8N (three mitotic divisions) (hereafter referred to as stages).

**Figure 1:**
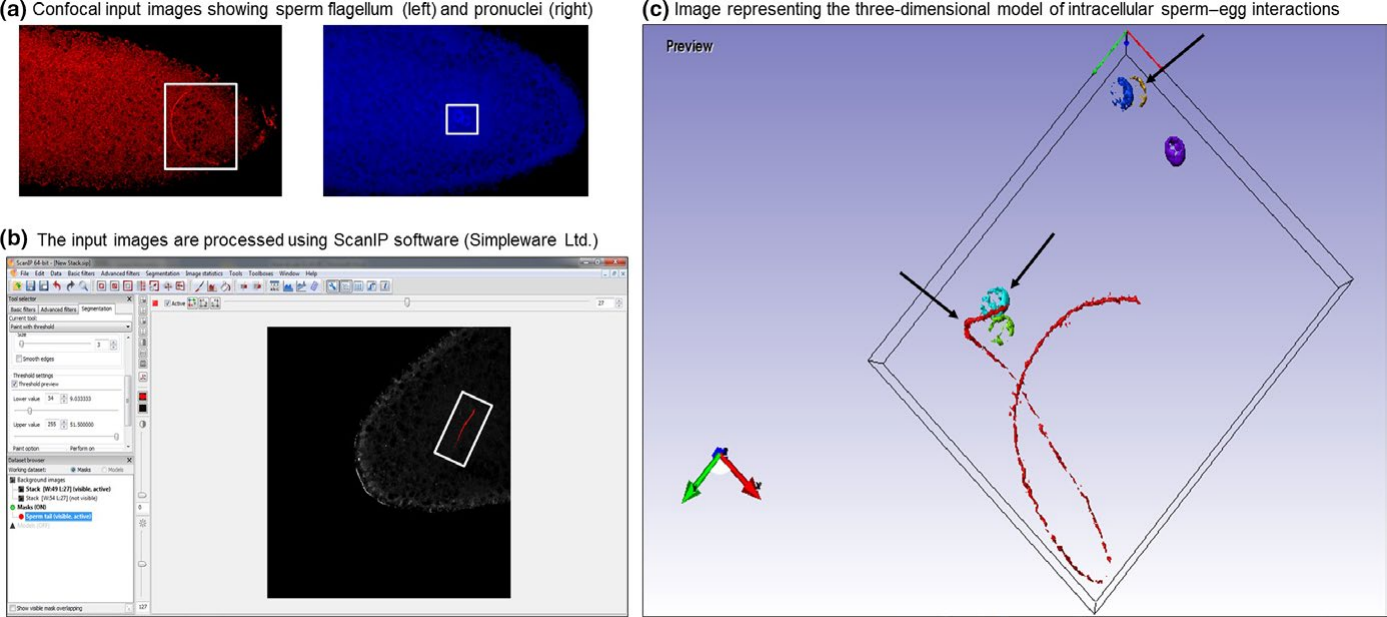
Overview of image processing. (a) Two confocal input images each showing a slice through the z‐stack of the egg where the sperm flagellum (left) and pronuclei (right) were present, (b) confocal images being processed using ScanIP. The image shown of the egg is one slice through the z‐stack where the sperm flagellum (red) was present, (c) the three‐dimensional model of intracellular sperm–egg interactions with (left to right) the sperm flagellum, pronuclei and polar bodies highlighted

For reconstructing a 3D image of the fertilized egg and subsequently measuring sperm flagellum shape, the confocal images were imported into Simpleware ScanIP (Synopsys, Mountain View, USA), a 3D image visualization and processing software (Figure 1). Spacing values for each stack of images were set to 0.002 mm to represent the images being taken at 2 μm through the z‐stack. The entire sperm flagellum and all pronuclei/nuclei and polar bodies (where present) were “masked” and reconstructed into a 3D model by the software. Each structure was assigned to one mask. For the sperm flagellum mask, we noted the general location of the sperm using the “paint with threshold” function and then reconstructed the sperm into a 3D model by the software. This function enables painting on the target area of the image with voxels of the resulting mask based on the image voxel intensity (i.e., voxels with intensity within a specific range of values are masked), and the nontarget items are ignored. A curve was then fitted to the sperm flagellum using a purpose built plug in for ScanIP which generated points along the sperm for subsequent analysis. To generate these points, an intensity value is assigned to each voxel in the mask, based on the number and distance of its neighbors. The voxel of highest intensity is taken, and the centroid of its neighbors is recorded as the starting point. From the starting point, nearby voxels are weighted based on distance, and intensity, and the neighborhood centroid of the voxel with greatest weight is taken as the second point. With the starting point as point 0 and the second point as point 1, the next point is determined iteratively in the same way, but with the direction from point 0 to point 1 taken into account and adjusting the weighting of each voxel (i.e., voxels behind the direction of iteration are not considered). The preferred step distance is also modified based on the angle of the considered point from the direction of iteration to make the step size adaptive to curvature. Iteration continues until the distance from the current end point to the next found point is below a minimal or no next point is found. As each point is found, the voxels between the found point and the previous point are removed from consideration which allows the algorithm to cope with a curve crossing over itself. The iterative process is then repeated in the opposite direction, taking the starting point as point 1 and the second point as point 0. The spacing between points on the sperm flagellum curve was automated to keep them consistent between samples. Thus, we had the coordinates of each point along the sperm which allowed fitting a curve so that we could compare sperm shape within developmental stages of the same species, between developmental stages of the same species, the same stage between species.

For polar body and pronuclear positions, each of those structures was assigned their own mask, and the area to be measured was again identified using the “paint with threshold” function. A purpose built plug in then takes the center positions of the painted voxels of the target and determines the simple arithmetic mean of each *x*,* y*,* z* coordinate to calculate the target center (e.g., for a particular target, if only two voxels were in the mask for that target (NB: there are many more than this), at positions [3,9, 7] and [5,3,7], then the center would be at [(3 + 5)/2, (9 + 3)/2, (7 + 7)/2] = [4,6,7]. Thus, we obtained single central positions for each of the pronuclei and polar bodies.

### 2.4 ISEI parameters and their measurements

We used the acquired *x*,* y*, and *z* coordinates to calculate seven *parameters* describing sperm shape and its position in the egg (see Appendix S1 for summary descriptions) To determine *sperm positioning* (mm) within the egg, we used the distance from the polar bodies to various points along the sperm flagellum as a landmark for where the sperm flagellum was positioned within the egg. Polar bodies are haploid cells that are formed during oogenesis (Tremblay & Caltagirone, 1973). One of the haploid nuclei becomes the female pronucleus and the other three, the polar bodies, fail to develop and migrate to the putative same position within the egg. As such, they potentially are a useful landmark for orientation of the sperm within the egg. We also calculated the three‐dimensional *distance between the male and female pronuclei* (mm). These analyses were performed using Python (2.7.10, 2015).

Several parameters have been developed to characterize the shape of filamentary structures. These have been employed in, for example, studies of DNA structure (Fuller, 1971), the mechanics of flagellum motion (Sartori, Geyer, Scholich, Jülicher, & Howard, 2016), as well as applications in nonbiological disciplines. We used these to quantify sperm flagellar shape inside the egg. *Arc length* (mm) measures the total length of the curve between the two ends of the sperm. *Net length* (mm) is the total 3D length of the sperm. *Aspect ratio* (dimensionless) gives the arc length divided by the net length. Thus, if the curve is straight, then the aspect ratio will be 1, but a wavy curve or a curve which moves back and forth will have a larger aspect ratio. C*urvature* (1/mm) as a function of position describes local deviation from straight lines. Imagine fitting a circle to the curve at any point. If the best fit circle has radius *r,* then the curvature at that point will be 1/*r*. Thus, tight circles have higher curvature. We calculated *average curvature* (or *curvature*) over the arc length of the flagellum but also determined *total curvature* as the net curvature summed over the flagellum. One may also consider the direction to the center of the best‐fit circle. This direction vector may also rotate. We can use this rotation to detect three‐dimensional structure such as helices. The *torsion* (1/mm) measures how much the curve locally resembles a helix, how tight the helix (curve) is wound, and whether the helix is right or left handed. We averaged the torsion over the length of the flagellum. *Writhe* (Fuller, 1971) provides an alternative measure of helical structure, as well as other shapes such as a figure 8. To calculate arc length, aspect ratio, curvature, torsion, and writhe, we used Mathematica (Wolfram Research, Inc., 2014). Unfortunately, calculations of torsion and writhe are more sensitive to small measurement error than the other measurements. To obtain curvature, one needs to find the acceleration (second derivative with respect to arc length) along the curve, but torsion and writhe require the rate of change (i.e., a third derivative) of the curvature direction. Each derivative increases the measurement error due to small fluctuations in the positions of points along a curve. Preliminary analyses indicated high variability of these two parameters within conspecific crosses (data not shown), so we did not use these for further analyses.

We analyzed different species and crosses between species across up to four stages of development (Table 1). When analysing the pronuclear (PN) stage specifically, we measured all seven ISEI parameters: sperm positioning within the egg, pronuclei distance, arc length, net length, aspect ratio, curvature, and total curvature. However, following karyogamy (2N–8N stages), pronuclei fuse and polar bodies disintegrate so can no longer be measured. Thus, for 2N–8N stages, we only calculate arc length, net length, aspect ratio, curvature, and total curvature. Table 1 shows that we only capture the PN stage in crosses involving BOG; this is simply because we were not interested in stages past this, given that BOG and PSE are subspecies with limited evidence of reproductive incompatibility. Table 1 also shows that we lack data on PER from the 2N stage; this is simply because, despite scanning hundreds of eggs in which we found embryos at more advanced stages, we did not find 2N embryos that we could image.

**Table 1:** Number of fertilized eggs analyzed for each cross and each developmental stage

Female	Male	PN	2N	4N	8N
Bog	Bog	10			
Pse	Pse	8	5	6	10
Per	Per	10		10	10
Mir	Mir	10	12	13	12
Bog	Pse	8			
Pse	Bog	8			
Per	Pse	8	4	10	9
Pse	Per	7	10	10	11

### 2.5 Statistical analyses

Using these data, we asked whether ISEIs vary: (1) across development within a species, (2) among species at the same developmental stage, and (3) between parental and reciprocal hybrids to determine the contribution of sperm, egg, and interactions between gametes ISEIs. All statistical tests were performed using the open source software package R 3.1.0 (R Development Core Team, 2014). We performed two types of analyses. For each parameter, to compare within a species across developmental stages or between species at specific developmental stages, we performed one‐way ANOVAs. To compare sperm parameters between hybrids and parental crosses to assess the extent to which sperm and/or eggs influence ISEIs, we performed two‐way ANOVAs (male origin, female origin, and their interaction) for each parameter. Post hoc analyses were performed on statistically significant results using a Tukey's Honest Significant Difference (HSD) test.

## 3 RESULTS

### 3.1 Do ISEIs vary across development within a species?

**Figure 2:**
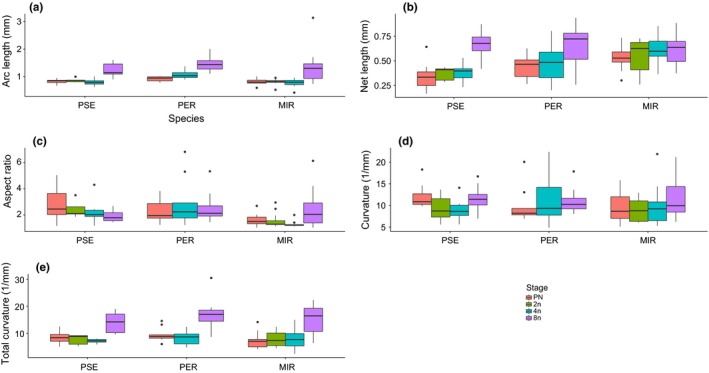
Within species comparison of ISEI parameters (±SE) from the PN to the 8N developmental stage. (a) Distance from pronuclei (mm) (“Distance”), (b) Sperm positioning (mm), (c) Average arc length (mm), (d) Net length (mm), (e) Average aspect ratio (dimensionless), (f) Average curvature (1/mm), and (g) Total curvature (1/mm). Bog = *D. ps. bogotana*, Pse = *D. ps. pseudoobscura*, Per = *D. persimilis*, and Mir = *D. miranda*

Sperm conformation has been suggested to change as the embryo develops (Pitnick & Karr, 1998). Here, we quantitatively test this hypothesis in three species (Table 2). For all three species, arc length (Figure 2a) and total curvature (Figure 2e) were significantly different across development. Net length (Figure 2b) significantly increased across zygotic development for two species (*D. pseudoobscura* and *D. persimilis*). Tukey HSD comparison for each species for these traits confirmed that substantial change occurred at the 8N stage compared to all earlier stages (Tables S2–S4). Aspect ratio also changed at the 8N stage for *D. miranda* (Figure 2c, Table 2; Tables S2–S4).

**Table 2:** Within species comparison of ISEI parameters from PN to 8N for three species (Pse, Per, Mir; *df* = degrees of freedom, based on number of developmental stages analyzed)

Trait	Pse (*df* = 3)	Per (*df* = 2)	Mir (*df* = 3)
Arc length	14.25, **<0.001**	21.38, **<0.001**	8.05, **<0.001**
Net length	13.39, **<0.001**	3.76, **0.036**	0.91, 0.44
Aspect ratio	1.58, 0.22	0.51, 0.61	4.13, **0.01**
Average Curvature	1.7, 0.19	0.3, 0.74	1.01, 0.39
Total curvature	11.83, **<0.001**	15.14, **<0.001**	10.72, **<0.001**

For each column, the first number is the *F*‐value, and second number is the *p*‐value. Significant comparisons in bold. Post hoc analyses were performed on statistically significant results using a Tukey's Honest Significant Difference (HSD) test reported in Table S1. See also Figure 2. Pse = *D. ps. pseudoobscura*, Per = *D. persimilis*, and Mir = *D. miranda*.

### 3.2 Do ISEIs differ between species at specific developmental stages?

**Figure 3:**
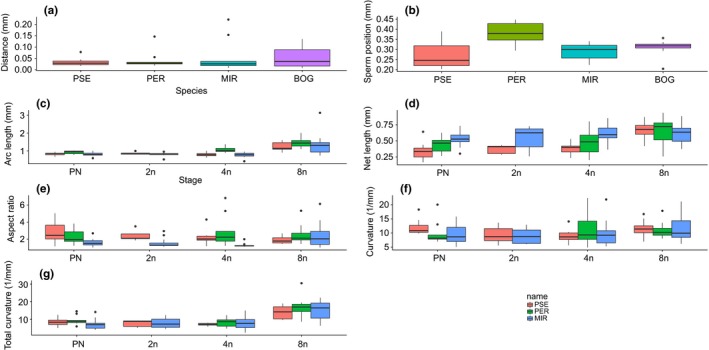
Between species comparison of ISEI parameters (±*SE*) at each developmental stage. (a) Distance from pronuclei (mm) (“Distance”), (b) Sperm positioning (mm), (c) Average arc length (mm), (d) Net length (mm), (e) Average aspect ratio (dimensionless), (f) Average curvature (1/mm), and (g) Total curvature (1/mm). Bog = *D. ps. bogotana*, Pse = *D. ps. pseudoobscura*, Per = *D. persimilis*, and Mir = *D. miranda*

We compared ISEIs among species at each developmental stage to assess whether species vary in these parameters (Table 3). We acquired data at only the PN stage for *D. pseudoobscura bogotana*, so compare them with the other three species for the two ISEI parameters of distance between pronuclei and sperm positioning. Sperm positioning differed (Figure 3b) with *D. persimilis* having larger average distances between the polar bodies and points along the sperm (Tables S2–S4). Some ISEI parameters differed among species at particular developmental stages, but this response was inconsistent across development (Figure 3, Table 3). The only ISEI trait that showed consistent differences across development was for net length in which *D. miranda* was longer than *D. pseudoobscura* up until the 8N stage (Figure 3d; Tables S2–S4).

**Table 3:** Between species comparison of ISEI parameters (±SE) at each developmental stage

Trait	PN (*df* = 3)	2N (*df* = 1)	4N (*df* = 2)	8N (*df* = 2)
PN distance	0.37, 0.78			
Sperm positioning	6.96, **<0.001**			
Arc length	1.47, 0.24	1.51, 0.30	13.72, **<0.001**	0.64, 0.54
Net length	3.63, **0.02**	6.03, **0.03**	4.37, **0.02**	0.28, 0.76
Aspect ratio	2.55, 0.07	7.22, **0.02**	5.37, **0.01**	0.84, 0.44
Average curvature	1.36,0.27	0.08, 0.78	0.45, 0.64	0.08, 0.93
Total curvature	1.26, 0.30	0.016, 0.9	0.32, 0.73	1.08, 0.35

*df* = degrees of freedom, which varies between developmental stage dependent on what species are being compared (PN comparisons include all four taxa—Pse, Per, Mir, and Bog; 2N compares Pse and Mir; 4N and 8N compares Pse, Per, and Mir). For each column, the first number is the *F*‐value, and second number is the *p*‐value. Significant comparisons in bold. Post hoc analyses were performed on statistically significant results using a Tukey's Honest Significant Difference (HSD) test reported in Table S1. See also Figure 3.

### 3.3 What are the relative roles of female origin, male origin, and their interaction in mediating ISEIs?

**Figure 4:**
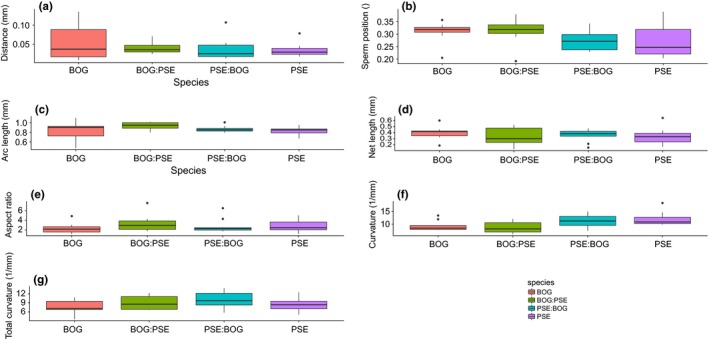
Parental (Bog, Pse) and hybrid comparison (Bog:Pse, Pse:Bog) of ISEI parameters at the PN stage (±*SE*). (a) Distance from pronuclei (mm) (“Distance”), (b) Sperm positioning (mm), (c) Average arc length (mm), (d) Net length (mm), (e) Average aspect ratio (dimensionless), (f) Average curvature (1/mm), and (g) Total curvature (1/mm). Bog = *D. ps. bogotana*, Pse = *D. ps. Pseudoobscura*. Female origin listed first

We compared parental and hybrid crosses to determine whether ISEIs are influenced by the species origin of either egg or sperm or is a result of interactions between the gametes (Table 4, Figure 4). For crosses between *D. ps. pseudoobscura* and *D. ps. bogotana*, we only measured at the PN stage. ISEI traits did not differ between eggs fertilized by either species with the exception of average curvature in which the two parental crosses differed from each other but not from either reciprocal hybrid cross (Figure 4; Tables S2–S4), suggesting hybrids took on an intermediate form compared to the parental forms.

**Table 4:** Parental and hybrid comparisons of ISEI parameters during development

Trait	Effect	PN: Pse, Bog	PN: Pse, Per	2N: Pse, Per	4N: Pse, Per	8N: Pse, Per
PN distance	F	1.01, 0.32	0.18, 0.68			
	M	0.62, 0.44	0.39, 0.54			
	F*M	0.20, 0.66	2.23, 0.15			
Sperm positioning	F	3.36, 0.08	5.61, **0.02**			
	M	0.01, 0.92	6.72, **0.01**			
	F*M	0.00, 0.99	2.16, 0.15			
Arc length	F	0.42, 0.52	1.34, 0.26	0.40, 0.54	5.95, **0.02**	4.48, **0.04**
	M	0.62, 0.44	3.07, 0.09	0.35, 0.56	9.69, **<0.01**	0.15, 0.70
	F*M	2.78, 0.11	3.13, 0.09	No data	1.97, 0.17	3.08, 0.09
Net length	F	0.21, 0.65	0.56, 0.46	0.001, 0.97	0.17, 0.68	3.18, 0.08
	M	0.75, 0.39	2.02, 0.17	1.68, 0.21	2.51, 0.12	2.37, 0.13
	F*M	0.27, 0.61	0.003, 0.96	No data	8.07, **0.01**	0.003, 0.96
Aspect ratio	F	0.01, 0.92	0.44, 0.51	0.02, 0.88	2.21, 0.15	6.79, **0.01**
	M	1.37, 0.25	0.97, 0.33	0.01, 0.93	0.42, 0.52	1.5, 0.22
	F*M	1.00, 0.33	0.15, 0.70	No data	7.46, **0.01**	1.66, 0.21
Average curvature	F	9.96, **<0.01**	1.59, 0.22	0.51, 0.49	2.61, 0.12	0.13, 0.72
	M	0.12, 0.73	0.56, 0.46	0.04, 0.85	0.52, 0.48	0.03, 0.87
	F*M	0.85, 0.36	0.10, 0.75	No data	0.64, 0.43	0.35, 0.56
Total curvature	F	1.78, 0.19	0.65, 0.43	0.01, 0.93	2.35, 0.14	1.98, 0.17
	M	0.00, 0.99	0.001, 0.97	0.08, 0.79	0.68, 0.42	0.71, 0.40
	F*M	3.47, 0.07	1.96, 0.17	No data	0.66, 0.42	0.46, 0.50

**Figure 5:**
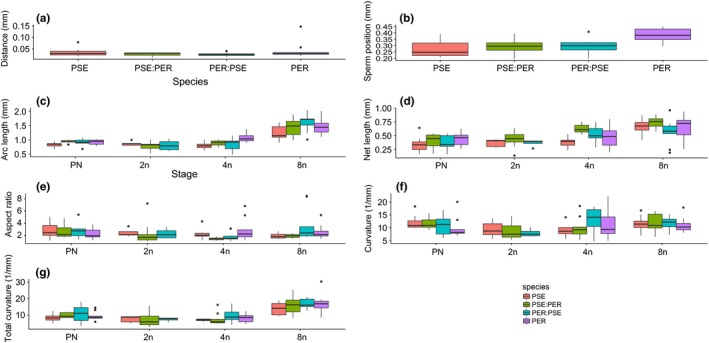
Parental (Pse, Per) and hybrid comparison (Pse:Per, Per:Pse) of ISEI (±*SE*). Parameters at each developmental stage. (a) Distance from pronuclei (mm) (“Distance”), (b) Sperm positioning (mm), (c) Average arc length (mm), (d) Net length (mm), (e) Average aspect ratio (dimensionless), (f) Average curvature (1/mm), and (g) Total curvature (1/mm). Pse = *D. ps. pseudoobscura*, Per = *D. persimilis*. Female origin listed first

Crosses between Pse and Bog were only performed at the PN stage, whereas crossed between Pse and Per were performed from PN to the 8N stage. Significant comparisons in bold. Post hoc analyses were performed on statistically significant results using a Tukey's Honest Significant Difference (HSD) test reported in Table S1. See also Figures 4 and 5.

For crosses between *D. ps. pseudoobscura* and *D. persimilis,* we measured ISEIs from the PN to 8N stage (Table 4, Figure 5). At the PN stage, only sperm position was influenced by both female and male gamete origin; eggs and sperm from *D. persimilis* resulted in greater sperm positioning, that is they have a larger average distance between the polar bodies and points along the sperm, than eggs and sperm from *D. pseudoobscura* (Figure 5b; Tables S2–S4; Appendix S1). Gamete origin influenced other ISEI parameters but at later developmental stages. For arc length, female origin had a consistent effect in which the parental crosses differed from each other but not from either reciprocal hybrid at 4N and 8N (Table 4; Figure 5c). Thus, the hybrids took an intermediate form from the parentals. Aspect ratio showed inconsistent effects of gamete origin on crosses (Figure 5e), and net length (Figure 5d) differed between crosses only at the 4N stage (Table 4; Tables S2–S4).

## 4 DISCUSSION

ISEIs are critical to successful karyogamy and subsequent development, yet sperm morphology rapidly evolves among species (see references in the Introduction). How this rapid evolution impacts ISEIs is not known so, here, we report on an analytical pipeline to quantify intracellular sperm and egg parameters and test whether these parameters change across development, differ among species, and—by utilizing hybrids between species—determine the relative contribution of sperm and egg to ISEIs. We quantified seven ISEI parameters, some measurable only during the pronuclear stage (pronuclei distance and sperm positioning) and others (arc length, net length, aspect ratio, average curvature, and total curvature) measurable throughout development where we analyzed to the 8N stage. While early *Drosophila* development is syncytial, ISEI parameters can be measured regardless of whether or not cell membranes are formed continuously in the developing embryo.

We quantitatively confirmed that, in conspecific crosses, sperm within an egg change morphology as development proceeds (Karr, 1996; Karr & Pitnick, 1996; Pitnick & Karr, 1998; Snook & Karr, 1998). In particular, substantial changes in arc length, net length, aspect ratio, and total curvature occurred at the 8N stage for all three species. Changes can occur if the sperm increases its size, or changes its shape, or both.

Note that, if a sperm grows larger but retains the same shape, then net length and arc length will both grow with the same fractional increase. On the other hand, aspect ratio and total curvature would remain constant, and average curvature (=total curvature/ arc length) would decrease. Table 2 shows that, in fact, average curvature does not decrease and total curvature increases, so at the 8N stage, across species, more folding of the sperm shape occurs.

This is still very early in development, prior to cytokinesis and cellularization. To our knowledge, there is no unique embryonic developmental process that occurs during the 3rd mitotic division. However, from the perspective of sperm behavior within the egg, the consistent response across species suggests something novel must be occurring. There are several putative molecular processes happening in early development that could contribute to these changes. Sperm proteins diffuse or are stripped from the *Drosophila* sperm flagellum throughout development (Pitnick & Karr, 1998; Politi et al., 2014). This includes the disintegration of the sperm plasma membrane that is initiated by endocytic and autophagic vesicles for paternal mitochondrial derivative destruction (PMD; Politi et al., 2014). While the flagellum is still largely intact 15–30 min after egg laying, the association of vesicles that begin PMD in *D. melanogaster* occurs around this time (Politi et al., 2014), which also corresponds to the timing of the 2nd to 3rd nuclear division (Tyler, 2000), when we observed these significant changes in sperm shape within the embryo. Perhaps PMD alters sperm conformation and, because we were tracking sperm shape with flagellum‐specific antibody labeling, we could begin to quantify the impact of PMD on this process. The remainder of the sperm tail ends up in the developing midgut and is defecated by the hatched larvae (Pitnick & Karr, 1998). Thus, there is an early, precellular and then subsequent interaction between components of the egg cytoplasm and interactions with the sperm flagellum.

Recent work has also identified that zygotic gene activity occurs much earlier in *Drosophila* embryonic development than previously appreciated, prior to the formation of the syncytial blastoderm (Ali‐Murthy, Lott, Eisen, & Kornbery, 2013). The finding of earlier transition from maternal to zygotic developmental control for at least some proportion of the genome was facilitated by improved detection methods (Lee et al., 2014). As these techniques continue to be developed, greater understanding of the molecular contribution of sperm in early embryogenesis may expand, contributing to understanding the evolution of ISEIs. This is critical because eggs not only receive a structural sperm payload that needs to be dealt with, but also sperm RNAs that are essential for fertilization and zygotic development; such structural and/or signaling factors from the sperm may complement maternal factors that contribute to early programming of embryonic development (Miller, 2015; Ntostis et al., 2017). Here, we show a consistent and substantial change in ISEIs at a specific developmental stage across several species. Future work could focus on this stage to help identify such early paternal and zygotic contributions to development.

Previous work, using *Drosophila* species that vary greatly in sperm length, has suggested that ISEIs differ between species (Karr & Pitnick, 1996). Here, we compared ISEI parameters between species that have similar sperm lengths across each developmental stage. We generally found similar shapes at each developmental stage among species. Given that the within‐species analysis, discussed above, showed substantial changes at the 8N stage, the lack of differences between species at the 8N stage indicates that species are responding similarly to whatever factors alter the interaction between the embryo and sperm structure at the 8N stage. Our results from hybrid crosses, while relatively few, suggest that changes in ISEIs are primarily mediated by the egg. This is not a particularly surprising result, given the primary role of maternal‐effect genes (mRNA and protein), accumulated during oogenesis, in early *Drosophila* development. Additionally, this may ensure that, while sperm can rapidly evolve in response to postcopulatory sexual selection, their primary function of karyogamy can be maintained. Thus, we find a relatively negligible male effect, but whether a larger paternal impact would be found in species where sperm length differed substantially between the female parental species and paternal parental species is unknown. The species analyzed here do not vary much in sperm length, and they are relatively short for *Drosophila* (Snook, 1997). Moreover, these species were relatively closely related to facilitate the generation of reciprocal hybrids. These traits were virtues while developing the methodology to quantify ISEIs but future work should study taxa with longer sperm and that are more evolutionary divergent to examine the effects of male and female origin on ISEIs, karyogamy, and development.

Our work shows proof of principle for quantifying ISEIs, examining the extent to which ISEIs change throughout development within a species, are different between species across development, and the influence of the male and female origin on ISEIs. To establish the method, we used a few *Drosophila* species that have relatively short sperm, are closely related, and tracked changes through the 8N stage. These criteria allowed us to confirm our measurements, but limit the wider evolutionary scope for the work. As *Drosophila* sperm persist in the embryo throughout development and species vary tremendously in sperm length, future work can capitalize on our foundation to describe (more complicated) ISEIs. Moreover, we also lay the foundation to quantify abnormal sperm conformation associated with early fertilization defects, arising from paternal and maternal mutations (Ohsako et al., 2003; Wilson et al., 2006). By describing sperm shape in fertilization events that are successful (as was done here) and comparing to shape to those that are not successful, but arise in a nonmutant background (i.e., unsuccessful hybrids after syngamy but prior to karyogamy), the role of sperm conformation in zygote viability can be tested.

While our work has focused on *Drosophila*, sperm morphology rapidly evolves in other taxa, and the whole (or a substantial portion of it) sperm enters the egg at syngamy, resulting in ISEIs (Karr et al., 2009; Loppin & Karr, 2005). These intracellular interactions between the gametes will be subject to natural selection as they are critical for successfully producing the next generation. Given the rapid evolution of sperm, determining whether these processes are highly conserved, and the extent to which either the male or female, or the interaction between the sexes, influences ISEIs will expand the understanding of the biology of reproduction and subsequent development. Additionally, most species do not have synticial development so the extent to which ISEIs are similar between *Drosophila* and species with cellularization immediately after karyogamy will need examining. This comparison could be important as increased knowledge of what aspects of ISEIs are either conserved or variable may have applied relevance. For example, the use of heterologous fertilization to combat infertility in endangered animals may be informed by the study of ISEIs (Karr et al., 2009). Even closely related species may not be a good match for heterologous fertilization; domestic cow oocytes were successfully fertilized by one species of *Oryx* (*O. demmah*) but not with another species (*O. gazelle callotis*; Kouba, Atkinson, Gandolf, & Roth, 2001). While some of the problems was unsuccessful syngamy, in successfully fertilized oocytes where karyogamy occurred, subsequent development was comprised. What explains this asymmetrical pattern between *Oryx* species, and whether structural species‐specific ISEIs may contribute, is unknown. However, if ISEIs do vary, it leaves the possibility that such information could be used to predict which species may be good matches for heterologous fertilization. Quantitative studies of ISEIs, such as that we present here, will further the understanding of fertilization and development, and studies performed across taxa will provide a basis for understanding the evolution of gametic interactions with the potential for applied relevance.

## CONFLICT OF INTEREST

The authors declare no conflict of interests.

## AUTHOR CONTRIBUTIONS

RRS conceived the study, HMS conducted the experiment under the guidance of RRS and PGY, HMS and MAB analyzed the data. HMS, MAB, and RRS interpreted the results. HMS, MAB, and RRS wrote the manuscript and PGY provided editorial advice.
