# Supplementary material for: Sperm morphology and the evolution of intracellular sperm–egg interactions
Source: Ecol Evol. 2018 Apr 24;8(10):5047–58. doi: 10.1002/ece3.4027 (PMC5980432; doi:10.1002/ece3.4027)
Supplement: Supplementary file 1 [file ECE3-8-5047-s001.pdf]

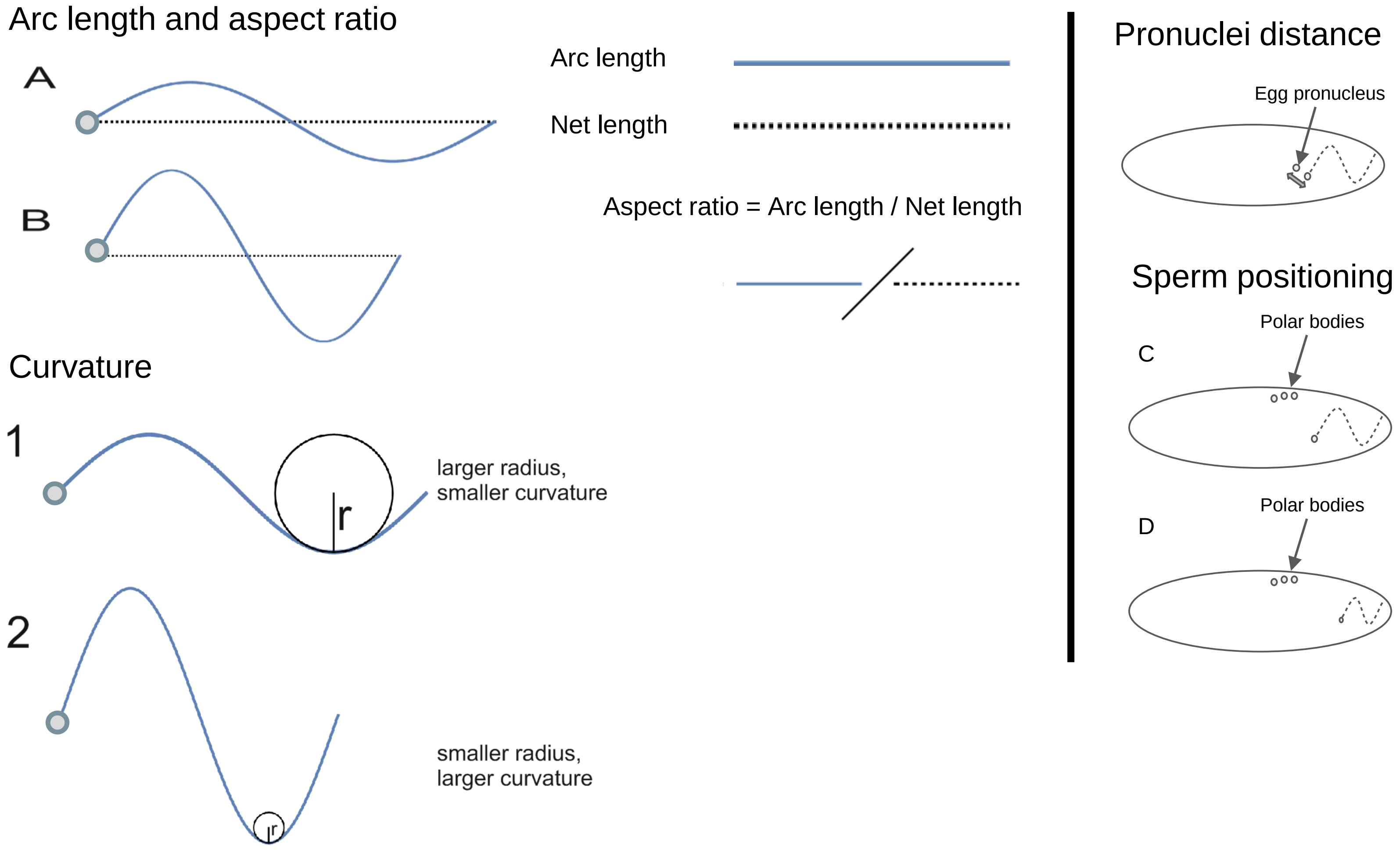

Arc length and aspect ratio
A
B
Arc length
Net length
Aspect ratio = Arc length / Net length
Curvature
1
larger radius,
smaller curvature
r
2
smaller radius,
larger curvature
r
Pronuclei distance
Egg pronucleus
Sperm positioning
C
Polar bodies
D
Polar bodies

Appendix 1: Representations of the sperm parameters measured.
Arc length gives the total length across the sperm flagellum and is the same for A) and B). Aspect ratio is arc length / net length and is higher in B) compared to A).

Average curvature is measured as an average across the entire flagellum length and is lower in 1) compared to 2). The schematic shows that the radius (r) of the circle in 1) is larger than the radius of the circle in 2) and as curvature is measured as 1/r, this gives the smaller value of curvature in 1) compared to 2).

Pronuclei distance is the distance (μm) between the pronuclei of the sperm and that of the egg. This is a three-dimensional measure. Sperm positioning (mm) is the distance between the average of the polar bodies and that of the average of equidistant points along the sperm flagella. In the example here, D) would have a higher value for sperm positioning than C).
